# Metformin inhibits proliferation and growth hormone secretion of GH3 pituitary adenoma cells

**DOI:** 10.18632/oncotarget.16556

**Published:** 2017-03-25

**Authors:** Jiayin An, Xiangdong Pei, Zhenle Zang, Zheng Zhou, Jintao Hu, Xin Zheng, Yin Zhang, Jiaojiang He, Lian Duan, Rufei Shen, Weihua Zhang, Feng Zhu, Song Li, Hui Yang

**Affiliations:** ^1^ Multidisciplinary Center for Pituitary Adenomas of Chongqing, Department of Neurosurgery, Xinqiao Hospital, Third Military Medical University, Chongqing, China; ^2^ Department of Neurosurgery, Lanzhou General Hospital of Chinese People's Liberation Army, Lanzhou, China; ^3^ Department of Endocrinology, Xinqiao Hospital, Third Military Medical University, Chongqing, China; ^4^ Department of Biology and Biochemistry, College of Natural Sciences and Mathematics, University of Houston, Houston, Texas, USA; ^5^ Innovative Drug Research Centre, University of Chongqing, Chongqing, China

**Keywords:** metformin, growth hormone-secreting pituitary adenoma, proliferation, ATF3, STAT3

## Abstract

Metformin is an anti-hyperglycemic agent used to treat diabetes, and recent evidence suggests it has antitumor efficacy. Because growth hormone-secreting pituitary adenoma (GH-PA) patients have a high incidence of diabetes frequently treated with metformin, we assessed the antitumor effect of metformin on GH-PA. We found that metformin effectively inhibited proliferation and induced apoptosis in the GH-PA cell line GH3. We detected a decrease in mitochondrial membrane potential (MMP), an increase in expression of pro-apoptotic proteins, and a decrease in expression of an anti-apoptotic protein in metformin-treated GH3 cells, which suggests involvement of the mitochondrial-mediated apoptosis pathway. Inhibition of AMPK, which is activated by metformin, failed to reverse the antiproliferative effect. ATF3 was upregulated by metformin, and its knockdown significantly reduced metformin-induced apoptosis. In addition, GH secretion was inhibited by metformin through suppression of STAT3 activity independently of AMPK. Metformin also significantly suppressed cellular proliferation and GH secretion in primary human GH-PA cells. Metformin also significantly inhibited GH3 cell proliferation and GH secretion *in vivo*. ATF3 upregulation and p-STAT3 downregulation were confirmed in xenografts. These findings suggest metformin is a potentially promising therapeutic agent for the treatment of GH-PA, particularly in patients with diabetes.

## INTRODUCTION

GH-PA is a common type of pituitary adenoma (PA). Although most GH-PAs are benign, they may cause patients to develop symptoms of acromegaly through hypersecretion of growth hormone (GH) [1]. The presence of excess GH and IGF-1 leads to a series of metabolic symptoms, including glucose intolerance (GI), diabetes mellitus (DM), hypertension, and several other endocrine disorders [2, 3]. If excess GH is sustained for a long period of time *in vivo*, the mortality rate may increase several fold, mainly due to cardiovascular complications [4, 5]. The treatments for GH-PA include surgery, medical management, and radiotherapy. Unfortunately, some invasive GH-PAs exhibit poor responses to these approaches [6]. Hence, it is necessary to develop novel treatment strategies.

Metformin is a safe and effective oral antidiabetic drug that is widely used clinically. Currently, it is the first-line drug used to treat type 2 diabetes (T2D) because it suppresses hepatic gluconeogenesis and promotes glucose uptake into skeletal muscles [7]. In addition to its glucose-lowering activity, there has recently been increasing interest in its antitumor potential. Many studies have reported the beneficial effects of metformin for cancer prevention and treatment [8, 9]. In addition, accumulating evidence indicates that metformin decreases cancer cell viability and tumor growth in xenograft models [10–12]. Recently, a short-term clinical study demonstrated that metformin reduces cellular proliferation *in vivo* in atypical endometrial hyperplasia and endometrial endometrioid adenocarcinoma [13]. Metformin is an activator of adenosine monophosphate-activated protein kinase (AMPK), which is a serine/threonine protein kinase. Its antitumor effects are mainly considered to occur through activation of AMPK [14]. It suppresses cancer cell growth by inducing cell cycle arrest and apoptosis in an AMPK-dependent manner [15]. However, it also triggers cell cycle arrest and apoptosis in an AMPK-independent mechanism in some tumors [16, 17].

The antitumor effects and mechanisms of metformin have been identified in many types of tumors; however, they are unknown in GH-PA. Because patients with GH-PA have a higher incidence of diabetes, which is the most well-known indication for the use of metformin, we explored the potential effects of metformin on the growth and GH secretion of GH-PAs *in vitro* using GH3 cell line, primary tumor cells and *in vivo* using nude mice. We also investigated the molecular mechanisms by which metformin exerts its effects.

## RESULTS

### Metformin inhibited GH-PA cell proliferation

**Figure 1:**
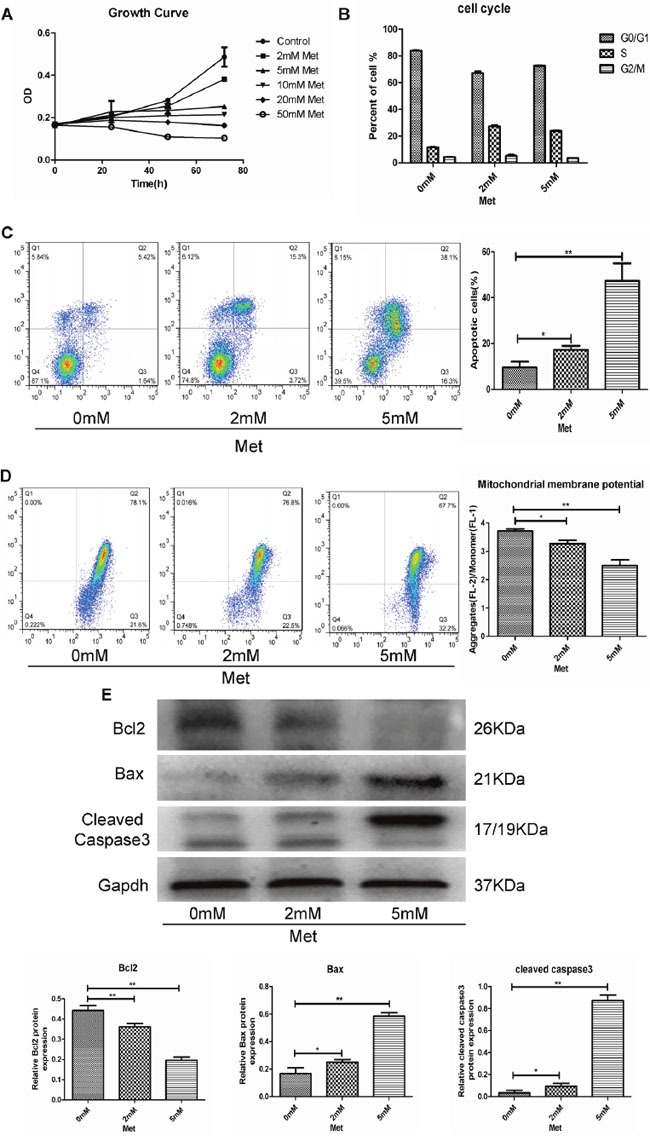
Metformin (Met) induced apoptosis via the mitochondrial apoptotic pathway in a dose dependent manner **(A)** GH3 cells were incubated with different concentrations of metformin for 0, 24, 48, and 72 h, and cell proliferation was measured by CCK-8 assay. **(B)** GH3 cells were exposed to 0, 2, and 5 mM metformin for 48 h, and then cell cycle was analyzed using flow cytometry. **(C)** Apoptosis of cells treated with metformin at different concentrations was measured by flow cytometry. **(D)** Mitochondrial membrane potential (MMP) was measured in different treatment groups by JC-1 staining. **(E)** Protein expression levels of Bcl2, Bax and Cleaved caspase3 were examined by Western blot. *P < 0.05;**P < 0.01.

First, we conducted Cell Counting Kit-8 (CCK-8) assays to investigate the effect of metformin on the proliferation of GH3 cells. GH3 cells were treated with different doses of metformin (0, 2, 5, 10, 20, and 50 mM) and were assessed at four time points (0, 24, 48 and 72 h). Growth curve analysis revealed that metformin inhibited GH3 proliferation in a dose-dependent manner (Figure 1A). Moreover, metformin effectively suppressed GH3 proliferation at dose as low as 0.2mM (Supplementry Figure 1A, 1B).

### Metformin induced apoptosis via the mitochondrial apoptotic pathway

**Figure 2:**
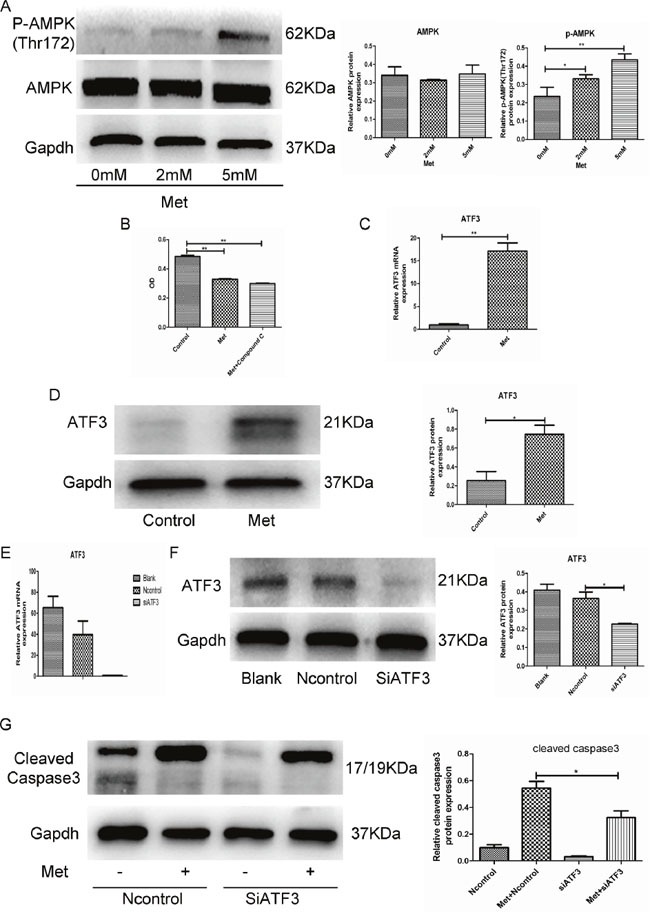
Metformin induced GH3 cell apoptosis by activation of ATF3 independent of AMPK **(A)** Protein levels of p-AMPK (Thr172) and AMPK were examined by Western blot. **(B)** GH3 cell proliferation with metformin treatment (5mM for 48 h) was assayed by CCK-8, following treatment with compound C (5 uM). **(C** and **D)** ATF3 mRNA and protein levels in 2 groups of treatment with or without metformin for 48 h by RT-qPCR and Western blot. **(E** and **F)** ATF3 mRNA and protein levels of cells transfected with control or ATF3 siRNA. **(G)** GH3 cells were transfected with control or ATF3 siRNA and then treated with 5 mM metformin for 48 h. Cleaved-caspase3 was examined by Western blot. *P < 0.05;**P < 0.01.

As metformin inhibited GH3 cell proliferation, we explored whether it also affected the cell cycle and apoptosis. GH3 cells were treated with different concentrations of metformin (0, 2, and 5 mM) for 48 h, and then cell cycle distributions and apoptosis were analyzed by flow cytometry. No dose-dependent changes in GH3 cell cycle progression were observed in response to the metformin concentrations assessed (Figure 1B and Supplementry Figure 2). However, the percentage of apoptotic cells was significantly increased in a dose-dependent manner (Figure 1C).

We performed JC-1 staining to detect changes in the mitochondrial membrane potential (MMP) in GH3 cells. We found that metformin decreased the MMP in a dose-dependent manner (Figure 1D). The expression of several mitochondrial apoptotic pathway-related proteins, including Bcl-2, Bax, and cleaved caspase-3(19), were determined by western blot analysis, which showed that the anti-apoptotic protein Bcl-2 was decreased by metformin in a dose-dependent manner. In contrast, the pro-apoptotic proteins Bax and cleaved caspase-3 were significantly increased in a dose-dependent manner (Figure 1E).

### ATF3 mediated metformin-induced GH-PA cell apoptosis

Metformin is a well-known activator of the AMPK signaling pathway. We found that metformin promoted AMPK phosphorylation in GH3 cells (Figure 2A). However, compound C, which is an AMPK pathway inhibitor, did not reverse the inhibitory effects of metformin (Figure 2B), indicating that metformin-induced apoptosis may be independent of the AMPK signaling pathway. Microarray analysis revealed that the RNA expression levels of many genes were altered in metformin-treated GH3 cells compared with control cells. Activating transcription factor 3 (ATF3), which was significantly upregulated (8-fold) in the metformin-treated GH3 cells (5 mM) and has been reported to be involved in cell apoptosis [20–23], was chosen for further analysis. Quantitative RT-PCR and Western blotting confirmed that the ATF3 mRNA and protein levels were significantly upregulated in the metformin-treated GH3 cells (Figure 2C, 2D). Next, we knocked down ATF3 expression in GH3 cells to investigate whether metformin-induced apoptosis is mediated by ATF3. ATF3 siRNAs were transfected into GH3 cells, resulting in significant downregulation of ATF3 at the mRNA and protein levels (Figure 2E, 2F). Further, ATF3 knockdown significantly suppressed the metformin-induced expression of cleaved caspase-3 (Figure 2G). These findings suggest that metformin-induced GH3 cell apoptosis is at least partially mediated by ATF3.

### Metformin inhibited GH secretion by GH-PA cells

**Figure 3:**
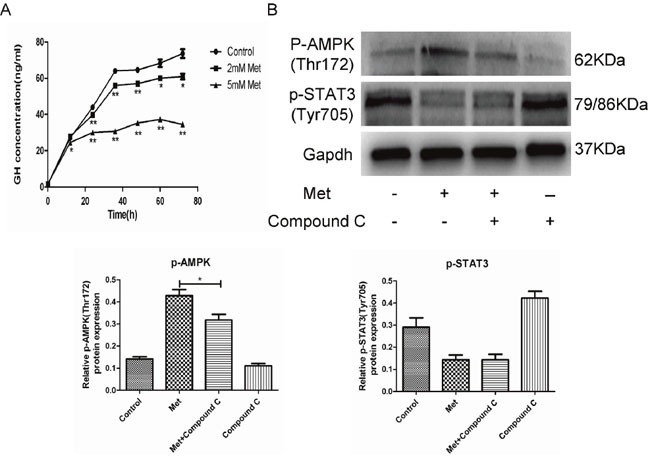
Metformin inhibited GH secretion by GH3 cells **(A)** GH3 cells were incubated with different concentrations of metformin for 0, 12, 24, 36, 48, 60 and 72 h, and GH secretion was detected by ELISA. **(B)** GH3 cells were pretreated with or without compound C and then treated with metformin. p-AMPK(Thr172) and p-STAT3(Tyr705) were examined by Western blot. *P < 0.05; **P < 0.01.

We next used ELISA to examine whether metformin inhibits GH secretion by GH3 cells. We found that GH release was significantly reduced by metformin in a dose and time-dependent manner (Figure 3A). GH hypersecretion by GH-PAs is induced in a STAT3-dependent manner [24]. We also found that STAT3 activity was suppressed by metformin. However, AMPK inhibition by compound C did not alter the inhibitory effect of metformin on STAT3 activity (Figure 3B). These results indicated that metformin inhibited GH secretion by GH3 cells through suppression of STAT3 activity in an AMPK-independent manner.

### Metformin inhibited GH-PA cell growth and GH secretion *in vivo*

**Figure 4:**
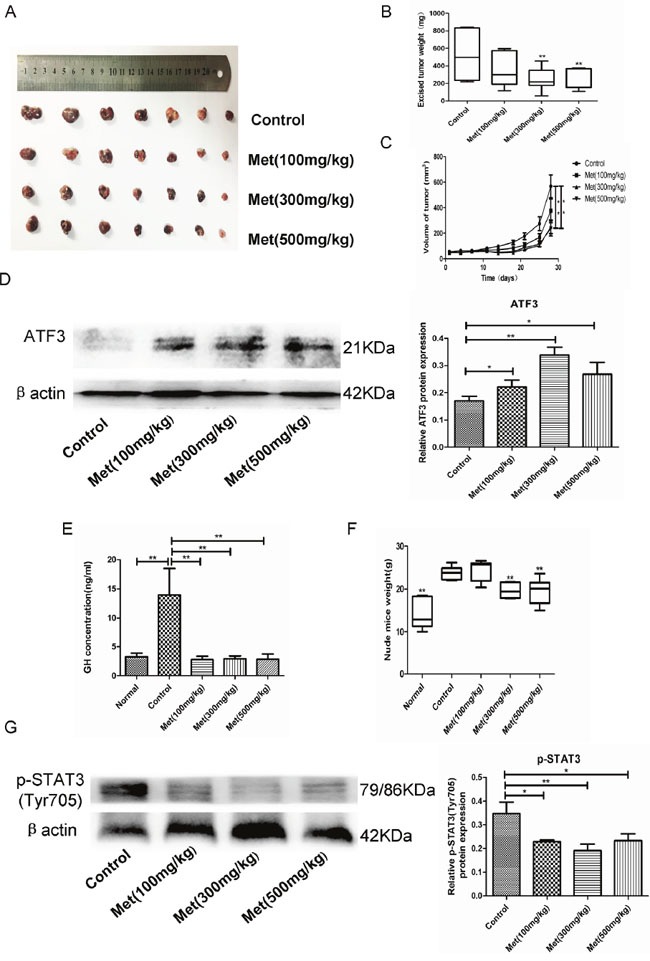
Metformin inhibited GH3 cell growth and GH secretion *in vivo* **(A)** GH3 cells were injected subcutaneously in nude mice and excised tumors from different groups are shown. **(B)** The weight of the excised tumors. **(C)** Growth curve shows the tumor volume change of mice after administration of vehicle or metformin. **(D)** Protein level of ATF3 in tumor tissues was examined by Western blot. **(E)** GH level of mice after different treatments was detected by ELISA. **(F)** The weight of the mice after different treatments was recorded. **(G)** Protein level of p-STAT3(Tyr705) in tumor tissues was examined by Western blot. *P < 0.05;**P < 0.01.

To further evaluate the antitumor potential of metformin *in vivo*, we generated a GH-PA xenograft model by subcutaneous injection of GH3 cells into nude mice. The mice were divided into normal and tumor-bearing groups. All mice were administered vehicle or metformin at one of 3 different concentrations (100 mg/kg, 300 mg/kg, or 500 mg/kg) each day for 4 weeks. Administration of metformin at the low concentration tended to non-significantly reduce tumor growth. In contrast, the middle and high metformin concentrations significantly inhibited tumor growth (Figure 4A, 4B, 4C). We also found that the tumor-bearing mice secreted more GH than the normal mice. Treatment of the tumor-bearing mice with metformin reversed GH hypersecretion (Figure 4E). In accordance with the clinical features of acromegaly patients, the body weights of the tumor-bearing mice were markedly increased compared with those of the normal mice due to GH secretion by the tumor cells. Metformin administration significantly attenuated the increased body weights of the tumor-bearing mice at the concentrations of 300 mg/kg and 500 mg/kg (Figure 4F). Moreover, the metformin-treated tumors exhibited increased ATF3 expression and reduced STAT3 phosphorylation, consistent with the *in vitro* results (Figure 4D, 4G).

### Metformin inhibited proliferation and GH secretion of patient-derived GH-PA cells

**Figure 5:**
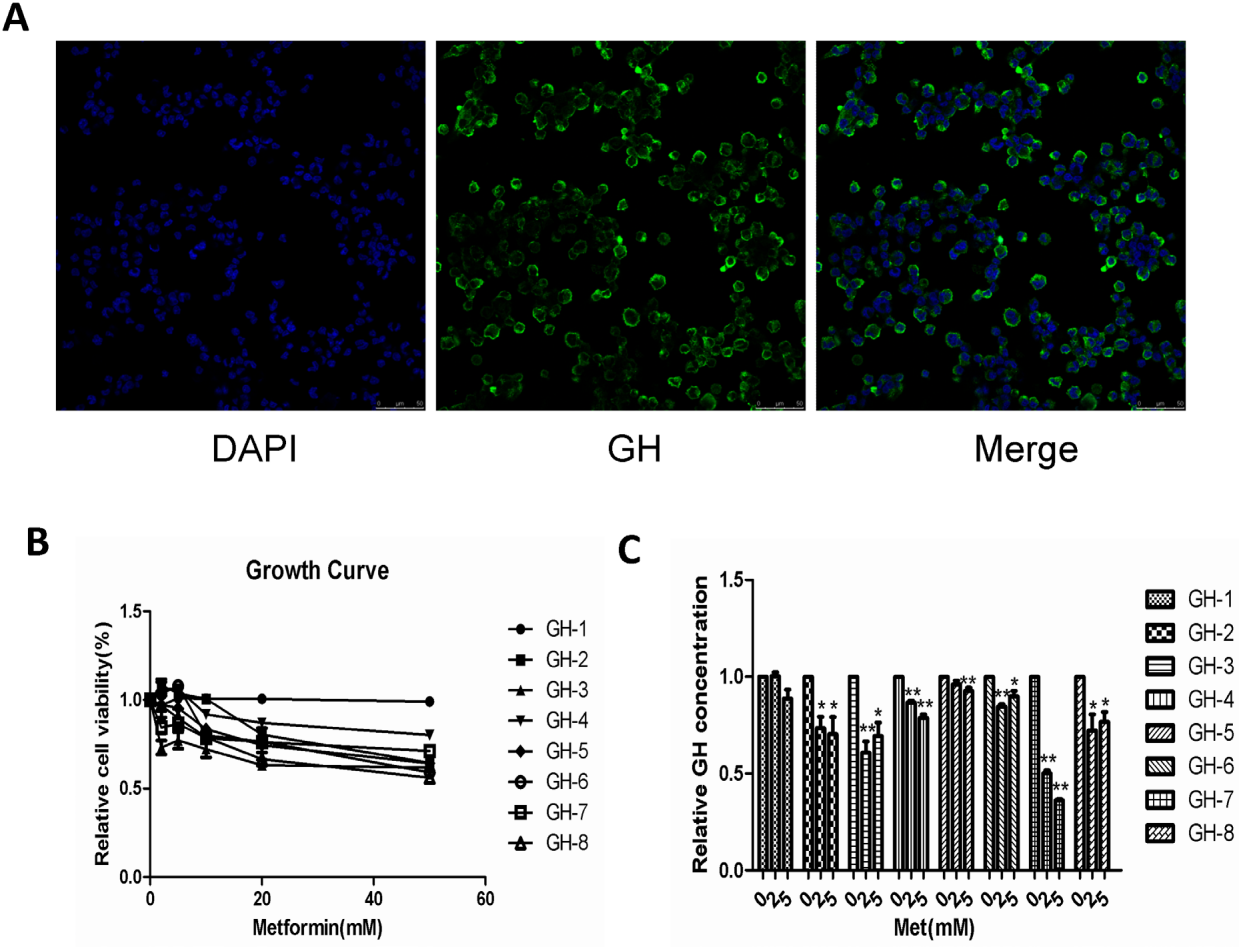
Metformin suppressed cellular proliferation and reduced GH secretion in primary cultures of human GH-PA cells **(A)** primary GH-PA cells were identified by human GH. Blue signal, DAPI nuclear staining; green signal, GH staining. Original magnification, ×400. **(B)** The effects of metformin on proliferation of the primary tumor cells, after 72 hours of exposure. **(C)** Detection of the primary cells GH secretion after treated with metformin by ELISA. *P < 0.05;**P < 0.01.

To further investigate the effects of metformin on human GH-PAs, we cultured and identified primary GH-PA cells derived from 8 patients after transsphenoidal resection (Figure 5A). Primary cultured cells were treated with different concentrations of metformin and assessed at 72 hours. Similar to results in GH3 cell line, metformin exerted growth inhibitory effects on 7 of the 8 individual patient cultures, whereas 1 of 8 samples did not respond (Figure 5B). Furthermore, a significant reduction of GH levels was detected in 7 of 8 tumor cell cultures after metformin treatment, while GH level was unchanged in 1 sample (Figure 5C). These data further verified the inhibitory effects of metformin on GH-PA.

## DISCUSSION

Although most GH-PAs are benign, some tumors exhibit invasive behaviors and respond poorly to current treatments. Thus, it is necessary to develop novel strategies to treat GH-PAs. In our study, we found that metformin inhibited the proliferation and hormone secretion of a GH-PA cell line both *in vitro* and *in vivo*.

A relatively non-toxic and well-tolerated antidiabetic drug, metformin also has antitumor potential, which has attracted increasing attention in recent years. Recently, studies have reported that metformin inhibits the growth of a number of different tumors [10–12]. The antitumor potential of this drug is not likely due to its reduction of blood glucose, as sulfonylureas and other antidiabetic drugs do not alter the risk of breast cancer [25]. Our study showed that metformin also inhibited proliferation of a GH-PA cell line, GH3, both *in vitro* and *in vivo*. Importantly, we further explored metformin's inhibition of GH-PA cell growth and found that primary cultured cells from 7 of 8 acromegaly patients responded to metformin treatment. A number of studies have suggested that the growth inhibitory effects of metformin may be attributed to cell cycle arrest or cell apoptosis. Ben Sahra et al. [26] demonstrated that metformin induces cell cycle arrest in the G0/G1 phase rather than promoting apoptosis in prostate cancer cells. However, metformin has also been reported to induce apoptosis in pancreatic cancer cells [27]. Our flow cytometry results indicated that metformin did not induce GH3 cell cycle arrest. PAs are senescent benign tumors, and Chesnokova et al. [28] and Arzt et al. [29] have reported these tumors were prematurely arrested in a state of cellular senescence which is the feature of stable maintenance of cell-cycle arrest. So, our finding that PA cell cycle was not arrested by metformin appears to be consistent with these studies. However, we found metformin significantly increased GH3 cell apoptosis in a dose-dependent manner. The discrepancy among studies regarding the effect of metformin may be attributed to the use of different cell types.

Apoptosis is programmed cell death triggered by intrinsic or extrinsic apoptotic pathways. The intrinsic pathway, also known as the mitochondrial-mediated pathway [30], is characterized by a decrease in the MMP and is regulated by Bcl-2 family proteins [31]. Among the Bcl-2 family members, Bax and Bcl-2 have been identified as the pivotal regulators of apoptosis. Pores form in the outer mitochondrial membrane in the presence of an increased Bax/Bcl-2 ratio. Consequently, apoptogenic mitochondrial proteins are released, activating downstream death programs, such as those involving caspase-3, and resulting in apoptosis [32]. Our results revealed that metformin significantly reduced the MMP of GH3 cells. Expression of the pro-apoptotic proteins Bax and cleaved caspase-3 was induced in the metformin-treated cells; however, metformin had an opposite effect on expression of the anti-apoptotic protein Bcl-2. Further, the Bax/Bcl-2 ratio was increased in response to metformin. These effects of metformin were dose dependent. Our findings suggest that metformin induces GH3 cell apoptosis through activation of the mitochondrial-mediated pathway.

AMPK, which has well-known antitumor effects, has been identified as the downstream molecule activated by metformin [14, 15]. In our study, AMPK was also activated in GH3 cells after metformin treatment *in vitro*. However, the AMPK inhibitor compound C did not reverse the anti-proliferative effect of metformin. These experimental results indicated that metformin inhibited GH3 cell proliferation in an AMPK-independent manner. To further identify the underlying molecular mechanisms of metformin-induced apoptosis, we conducted microarray analysis of GH3 cells treated (or untreated) with metformin. Gene expression profiles were significantly altered in GH3 cells following treatment with metformin, and in particular, ATF-3 expression was significantly upregulated (8-fold). We further demonstrated that ATF-3 was upregulated in metformin-treated GH3 cells at both the mRNA and protein levels; similar ATF-3 upregulation was detected in xenograft tumors from the metformin-treated nude mice. ATF3 is a stress-responsive transcription factor that belongs to the ATF/CREB family [33]. Its overexpression has been shown to induce apoptosis in both human ovarian surface epithelial and ovarian cancer cells [21]. ATF3 knockout partially reverses stress-induced beta-cell apoptosis [23]. ATF-3 is a key mediator of KLF6-induced apoptosis in prostate cancer cells [22]. In addition, it mediates apoptosis induced by t10, c12-CLA in human colorectal cancer cells [20]. Above all, ATF-3 plays a crucial role in regulating apoptosis. In the present study, ATF-3 knockdown using siATF-3 resulted in a marked decrease in metformin-induced apoptosis, suggesting that ATF-3 mediates the pro-apoptotic effect of metformin.

Further, we were interested in determining whether metformin affects GH secretion. Our results showed that metformin decreased GH secretion of GH3 cells both *in vitro* and *in vivo*. Metformin also suppressed GH secretion by primary human GH-PA cells. Additionally, the body weights of the tumor-bearing mice were markedly increased compared with those of the normal mice, and the metformin treatment reduced the body weights of the tumor-bearing animals in a dose-dependent manner over the course of the treatment. These findings indicated that metformin inhibited GH secretion *in vivo*. As an important energy-sensing enzyme, AMPK activity is closely related to hypophyseal hormone secretion. AMPK regulates secretion of luteinizing hormone (LH) in gonadotrope cells, as well as adrenocorticotropin precursor expression in corticotroph cells [34, 35]. However, a previous study has confirmed that AICAR (a specific AMPK agonist) does not affect hormone secretion by GH3 cells [36]. It has been recently reported that GH hypersecretion by GH-PAs is promoted by the transcription factor STAT3 [24, 37]. Based on these findings, we determined that metformin inhibited STAT3 activity both *in vitro* and *in vivo*. Therefore, our data suggest that the metformin might inhibit GH secretion by suppressing STAT3 activity independent of AMPK. Lin et al. [38] have also found that metformin suppresses STAT3 activation in an AMPK-independent manner in lung cancer, which is consistent with our findings.

In summary, we revealed that metformin inhibited somatotroph adenoma cell growth through an ATF-3-mediated pro-apoptotic effect both *in vitro* and *in vivo*. In addition, GH secretion was suppressed by metformin through inhibition of STAT3 signaling in an AMPK-independent manner. Our results suggest that metformin is a promising potential therapeutic agent for patients with benign GH-PA, especially those with diabetes. However, the results of this study must be verified by further clinical investigations.

## MATERIALS AND METHODS

### Cell culture

The rat pituitary adenoma cell line GH3 was obtained from American Type Culture Collection (ATCC, Manassas, VA, USA) and cultured in Ham's F-12K medium (Invitrogen Life Technologies, Carlsbad, CA, USA) supplemented with 2.5% fetal bovine serum (FBS; Invitrogen Life Technologies) and 15% horse serum (HS; Invitrogen Life Technologies) in a 5% CO_2_-humidified atmosphere at 37°C.

### Primary culture of human GH-PA cells

Eight human GH-PA specimens were obtained from patients undergoing transsphenoidal surgery at Xinqiao Hospital in Chongqing, China (Supplementry Table 1). All the participants voluntarily joined this study with informed consents. The human tumor tissues were obtained and used in a manner compliant with the Declaration of Helsinki. Fresh tumors were washed with 1X phosphate buffered saline and cut into small pieces. Then, tissue fragments were digested with Type I Collagenase for two hours at 37°C. After adding equal amounts of 10% FBS-containing MEM media, the cell suspension was filtered through 200 Mo filter. Then, the cell suspension was centrifuged and washed with PBS two times, and the cell pellet was resuspended in 10% FBS-containing MEM media. Finally, the primary cells were cultured in a 5% CO_2_-humidified atmosphere at 37°C.

**Table 1:** Primer list for qPCR

Gene name (Species)	Primer sequences (5’-3’)	Tm cycles	Product (bp)
ATF3 (rat)	F: CTCCTGGGTCACTGGTGTTTG	57-30	106
	R: GAGGACATCCGATGGCAAAG		
β-actin (rat)	F: GAGGGAAATCGTGCGTGAC	57-30	157
	R: GCATCGGAACCGCTCATT		

### Reverse transcription and qPCR

Total RNA was extracted using TRIzol Reagent (Invitrogen, Carlsbad, CA, USA) and then treated with DNase I to eliminate genomic DNA contamination. Reverse transcription of 1 ug RNA was performed using a ReverTra Ace First Strand cDNA Synthesis Kit (Toyobo, Osaka, Japan) in a final volume of 20 uL qPCR was performed using SYBR Premix Ex Taq II (TaKaRa, Dalian, China) and a CFX96 Real-time System (Bio-Rad Laboratories, Hercules, CA, USA). The relative expression levels were calculated using the 2^-ΔΔct^ method. The primer sequences used for qPCR are given in Table 1.

### Western blot analysis

GH3 cell extracts equivalent to 40 ug protein were subjected to 12% SDS-PAGE and then transferred onto polyvinylidene difluoride membranes. The membranes were blocked with 5% nonfat milk in Tris-buffered saline containing 0.05% Tween 20, incubated with rabbit antibodies against rat Bcl-2 (1:1000;Abcam), Bax (1:1000; Abcam), Caspase 3 (1:1000; CST), AMPK (1:1000; CST), p-AMPK(Thr172) (1:1000; CST), STAT3 (1:1000; CST), p-STAT3(Tyr705) (1:1000; CST), ATF3 (1:500; Proteintech), GAPDH (1:10000; Abcam) and β-actin (1:1000; CST). The membranes were further incubated with horseradish peroxidase-conjugated goat anti-rabbit IgG (1:2000; Santa Cruz Biotechnology). The membrane signals were visualized by enhanced chemiluminescence.

### Small interfering RNA (siRNA) experiments

SiRNA for rat ATF3 and control siRNA were purchased from Ruibo Biotech (Guangzhou, China). GH3 cells were transiently transfected with ATF3 siRNA and control RNA using riboFECT™ CP Reagent (Ribobio, Guangzhou, China) according to the manufacturer's instructions. Total RNA and protein were obtained from siRNA-transfected GH3 cells. siRNA transfection efficiency was determined by real-time PCR and Western blot.

### Cell proliferation assay

Cell proliferation was measured using a WST-8 Cell Counting Kit-8 (Dojindo Laboratories, Mashiki-machi, Kumamoto, Japan) according to the manufacturer's instructions.

### Cell cycle analysis

Cell cycle distribution was analyzed by flow cytometry. In total, 1.0×10^6^ cells were harvested at the indicated time points under the appropriate treatment. Then, cells were rinsed with PBS and fixed in 70% ethanol at 4 °C overnight. Subsequently, the cells were resuspended and stained in 0.05 mg/ml propidium iodide (PI; BD Biosciences Pharmingen) and analyzed by flow cytometry (FACScan; BD Biosciences Pharmingen, San Diego, CA, USA). Gating was set to exclude cell debris, cell doublets, and cell clumps. DNA histograms were analyzed by ModFit LT V2.0 software.

### Apoptosis analysis

GH3 cells were treated at the indicated time points with the appropriate treatment. After treatment, apoptosis was assessed using a FITC-Annexin V apoptosis detection kit (BD Biosciences Pharmingen). Cells were washed 3 times with PBS and suspended in binding buffer. Then, the cells were stained with FITC-Annexin V and PI. Finally, apoptosis was detected by flow cytometry and analyzed by ModFit LT V2.0 software.

### Microarray analysis in GH3 cells treated and untreated by metformin

Total RNA was extracted from GH3 cells using RNAiso Plus (Takara) and digested with DNase I to remove remaining DNA. The RNA was cleaned up with RNeasy Kit (Qiagen, Hilden, Germany) and the quantities and qualities were determined by spectrophotometry and 1% formaldehyde denaturing gel electrophoresis. The samples with bright bands of 28S to18S RNA in a ratio >1.5:1 were used for microarray analysis. The hybridization was performed using Affymetrix GeneChip® Rat Genome 230 2.0 Array. After hybridization, the GeneChip arrays were washed and then stained with streptavidin phycoerythrinonan (SAPE) with Affymetrix Fluidics Station 450 followed by scanning with the Affymetrix GeneChip Scanner 3000 7G. Differentially expressed genes were identified by significant analysis of microarray (SAM). Relative expression of ≥2.0 or ≤0.5 was considered as differentially expressed genes.

### Analysis of mitochondrial membrane potential (MMP)

MMP was determined by flow cytometry using the mitochondrial membrane potential assay kit with JC-1. Briefly, 1.0×10^6^ cells were harvested at the indicated time points with the appropriate treatment. Then, the collected cells were treated according to the manufacturer's instructions. Each sample was assessed by flow cytometry for red (JC-1 aggregates) and green (JC-1 monomers) fluorescence. JC-1 emits red fluorescence when it enters intact mitochondria to form aggregates. However, under the condition of MMP collapse, JC-1 forms a monomer and gives green fluorescence. So, the ratio of aggregates/monomer reflects the change of MMP.

### Enzyme-linked immunosorbent assay (ELISA)

The levels of GH were measured in the cell culture supernatants and plasma using ELISA kits (Invitrogen Life Technologies) at the indicated time points with the appropriate treatment according to the manufacturer's recommendations.

### *In vivo* tumorigenicity experiments

For the *in vivo* xenograft study, 4-week-old female BALB/cA-nu mice were purchased from Beijing HFK Bioscience Co., Ltd.(Beijing, China). Nude mice were housed under specific pathogen-free conditions. A total of 3×10^6^ GH3 cells suspended in 100 ul solution (50%PBS and 50% Matrigel), which had been washed twice and counted previously, were subcutaneously inoculated into the flank region of the mice. Treatment with metformin was started 9 days after inoculation. Then, the tumor bearing animals were randomly divided into 4 groups(7 mice/group) and 7 female nude mice without implantation with GH3 cells were used as a negative control group(normal group). The metformin-treated group received daily intragastric administration of 100, 300, or 500 mg/kg metformin for the next 4 weeks until the mice were sacrificed, while the control group and the normal group received daily intragastric administration of an equal volume of vehicle (H_2_O) only. The mice were monitored daily for any discomfort and weighed every two days to check for physical condition. Tumor volume was measured twice a week and calculated using the formula V (mm^3^) = [ab^2^]/2, where a is the length and b is the width of the tumor. Tumor tissue was removed from tumor-bearing nude mice and blood was collected following the final treatment. The plasma was stored at – 80°C until GH hormone assay as previously described. The study was approved by the Xinqiao Hospital institutional committee on animal care. All animal procedures were conducted according to protocols approved by UNC-CH Institutional Animal Care and Use Committee (IACUC).

Based on the well-established Reagan-Shaw method [18], the dose of drugs used for one animal species can be translated to another. According to the formula, human equivalent dose (mg/kg) = animal dose (mg/kg) × animal Km/human Km. The Km values are based on body surface area. Km for a 60 kg human adult is 37 and for a 20 g mouse is 3. Thus, the human equivalent of the dose of 500 mg/kg in a mouse is 2432 mg in an average-sized person of 60 kg, which is lower than the maximum safe dose of 2550 mg/d recommended in the Physician's Desk Reference. Thus, all dosing in our experiments were within a therapeutic range safely for humans.

### Statistical analysis

The data are expressed as the means ± SEM. Student t test, Mann–Whitney U test and one-way analysis of variance were applied to determine statistical significance. Data analysis were conducted by SPSS for Windows version 13.0 (SPSS Inc, Chicago, IL, USA). P values less than 0.05 were considered significant.

## SUPPLEMENTARY MATERIALS FIGURES



## References

[1] Schneider HJ, Sievers C, Saller B, Wittchen HU, Stalla GK (2008). High prevalence of biochemical acromegaly in primary care patients with elevated IGF-1 levels. Clin Endocrinol.

[2] Rosario PW (2011). Frequency of acromegaly in adults with diabetes or glucose intolerance and estimated prevalence in the general population. Pituitary.

[3] Melmed S (2006). Medical progress: acromegaly. N Engl J Med.

[4] Damjanovic SS, Neskovic AN, Petakov MS, Popovic V, Vujisic B, Petrovic M, Nikolic-Djurovic M, Simic M, Pekic S, Marinkovic J (2002). High output heart failure in patients with newly diagnosed acromegaly. Am J Med.

[5] Bihan H, Espinosa C, Valdes-Socin H, Salenave S, Young J, Levasseur S, Assayag P, Beckers A, Chanson P (2004). Long-term outcome of patients with acromegaly and congestive heart failure. J Clin Endocrinol Metab.

[6] Di Ieva A, Rotondo F, Syro LV, Cusimano MD, Kovacs K (2014). Aggressive pituitary adenomas--diagnosis and emerging treatments. Nat Rev Endocrinol.

[7] Shaw RJ, Lamia KA, Vasquez D, Koo SH, Bardeesy N, Depinho RA, Montminy M, Cantley LC (2005). The kinase LKB1 mediates glucose homeostasis in liver and therapeutic effects of metformin. Science.

[8] Tseng CH (2016). Metformin may reduce oral cancer risk in patients with type 2 diabetes. Oncotarget.

[9] Wan G, Yu X, Chen P, Wang X, Pan D, Wang X, Li L, Cai X, Cao F (2016). Metformin therapy associated with survival benefit in lung cancer patients with diabetes. Oncotarget.

[10] Quinn BJ, Dallos M, Kitagawa H, Kunnumakkara AB, Memmott RM, Hollander MC, Gills JJ, Dennis PA (2013). Inhibition of lung tumorigenesis by metformin is associated with decreased plasma IGF-I and diminished receptor tyrosine kinase signaling. Cancer Prev Res.

[11] Checkley LA, Rho O, Angel JM, Cho J, Blando J, Beltran L, Hursting SD, DiGiovanni J (2014). Metformin inhibits skin tumor promotion in overweight and obese mice. Cancer Prev Res.

[12] Zhou X, Chen J, Yi G, Deng M, Liu H, Liang M, Shi B, Fu X, Chen Y, Chen L, He Z, Wang J, Liu J (2016). Metformin suppresses hypoxia-induced stabilization of HIF-1α through reprogramming of oxygen metabolism in hepatocellular carcinoma. Oncotarget.

[13] Sivalingam V, McVey R, Gilmour K, Ali S, Roberts C, Renehan A, Kitchener H, Crosbie E (2015). A presurgical window-of-opportunity study of metformin in obesity-driven endometrial cancer. Lancet.

[14] Zakikhani M, Dowling R, Fantus IG, Sonenberg N, Pollak M (2006). Metformin is an AMP kinase-dependent growth inhibitor for breast cancer cells. Cancer Res.

[15] Queiroz EA, Puukila S, Eichler R, Sampaio SC, Forsyth HL, Lees SJ, Barbosa AM, Dekker RF, Fortes ZB, Khaper N (2014). Metformin induces apoptosis and cell cycle arrest mediated by oxidative stress, AMPK and FOXO3a in MCF-7 breast cancer cells. PLoS One.

[16] Malki A, Youssef A (2011). Antidiabetic drug metformin induces apoptosis in human MCF breast cancer via targeting ERK signaling. Oncol Res.

[17] Ben Sahra I, Regazzetti C, Robert G, Laurent K, Le Marchand-Brustel Y, Auberger P, Tanti JF, Giorgetti-Peraldi S, Bost F (2011). Metformin, independent of AMPK, induces mTOR inhibition and cell-cycle arrest through REDD1. Cancer Res.

[18] Reagan-Shaw S, Nihal M, Ahmad N (2008). Dose translation from animal to human studies revisited. FASEB J.

[19] Martinou JC, Youle RJ (2011). Mitochondria in apoptosis: Bcl-2 family members and mitochondrial dynamics. Dev Cell.

[20] Kim KJ, Lee J, Park Y, Lee SH (2015). ATF3 mediates anti-cancer activity of trans-10, cis-12-conjugated linoleic acid in human colon cancer cells. Biomol Ther.

[21] Syed V, Mukherjee K, Lyons-Weiler J, Lau KM, Mashima T, Tsuruo T, Ho SM (2005). Identification of ATF-3, caveolin-1, DLC-1, and NM23-H2 as putative antitumorigenic, progesterone-regulated genes for ovarian cancer cells by gene profiling. Oncogene.

[22] Huang X, Li X, Guo B (2008). KLF6 induces apoptosis in prostate cancer cells through up-regulation of ATF3. J Biol Chem.

[23] Hartman MG, Lu D, Kim ML, Kociba GJ, Shukri T, Buteau J, Wang X, Frankel WL, Guttridge D, Prentki M, Grey ST, Ron D, Hai T (2004). Role for activating transcription factor 3 in stress-induced beta-cell apoptosis. Mol Cell Biol.

[24] Zhou C, Jiao Y, Wang R, Ren SG, Wawrowsky K, Melmed S (2015). STAT3 upregulation in pituitary somatotroph adenomas induces growth hormone hypersecretion. J Clin Invest.

[25] Bodmer M, Meier C, Krahenbuhl S, Jick SS, Meier CR (2010). Long-term metformin use is associated with decreased risk of breast cancer. Diabetes Care.

[26] Ben Sahra I, Laurent K, Loubat A, Giorgetti-Peraldi S, Colosetti P, Auberger P, Tanti JF, Le Marchand-Brustel Y, Bost F (2008). The antidiabetic drug metformin exerts an antitumoral effect *in vitro* and *in vivo* through a decrease of cyclin D1 level. Oncogene.

[27] Wang LW, Li ZS, Zou DW, Jin ZD, Gao J, Xu GM (2008). Metformin induces apoptosis of pancreatic cancer cells. World J Gastroenterol.

[28] Chesnokova V, Zonis S, Kovacs K, Ben-Shlomo A, Wawrowsky K, Bannykh S, Melmed S (2008). p21(Cip1) restrains pituitary tumor growth. Proc Natl Acad Sci U S A.

[29] Arzt E, Chesnokova V, Stalla GK, Melmed S (2009). Pituitary adenoma growth: a model for cellular senescence and cytokine action. Cell Cycle.

[30] Takeda K, Stagg J, Yagita H, Okumura K, Smyth MJ (2007). Targeting death-inducing receptors in cancer therapy. Oncogene.

[31] Sprick MR, Walczak H (2004). The interplay between the Bcl-2 family and death receptor-mediated apoptosis. Biochim Biophys Acta.

[32] Adams JM, Cory S (1998). The Bcl-2 protein family: arbiters of cell survival. Science.

[33] Hai T, Wolford CC, Chang YS (2010). ATF3, a hub of the cellular adaptive-response network, in the pathogenesis of diseases: is modulation of inflammation a unifying component?. Gene Expr.

[34] Iwasaki Y, Nishiyama M, Taguchi T, Kambayashi M, Asai M, Yoshida M, Nigawara T, Hashimoto K (2007). Activation of AMP-activated protein kinase stimulates proopiomelanocortin gene transcription in AtT20 corticotroph cells. Am J Physiol Endocrinol Metab.

[35] Lu M, Tang Q, Olefsky JM, Mellon PL, Webster NJ (2008). Adiponectin activates adenosine monophosphate-activated protein kinase and decreases luteinizing hormone secretion in LbetaT2 gonadotropes. Mol Endocrinol.

[36] Tulipano G, Giovannini M, Spinello M, Sibilia V, Giustina A, Cocchi D (2011). AMP-activated protein kinase regulates normal rat somatotroph cell function and growth of rat pituitary adenomatous cells. Pituitary.

[37] Yu H, Jove R (2004). The STATs of cancer--new molecular targets come of age. Nat Rev Cancer.

[38] Lin CC, Yeh HH, Huang WL, Yan JJ, Lai WW, Su WP, Chen HH, Su WC (2013). Metformin enhances cisplatin cytotoxicity by suppressing signal transducer and activator of transcription-3 activity independently of the liver kinase B1-AMP-activated protein kinase pathway. Am J Respir Cell Mol Biol.

