# Supplementary material for: Metformin inhibits proliferation and growth hormone secretion of GH3 pituitary adenoma cells
Source: Oncotarget. 2017 Mar 25;8(23):37538–49. doi: 10.18632/oncotarget.16556 (PMC5514928; doi:10.18632/oncotarget.16556)
Supplement: Supplementary file 1 [file oncotarget-08-37538-s001.pdf]

Metformin inhibits proliferation and growth hormone secretion of GH3 pituitary adenoma cells

SUPPLEMENTARY MATERIAL

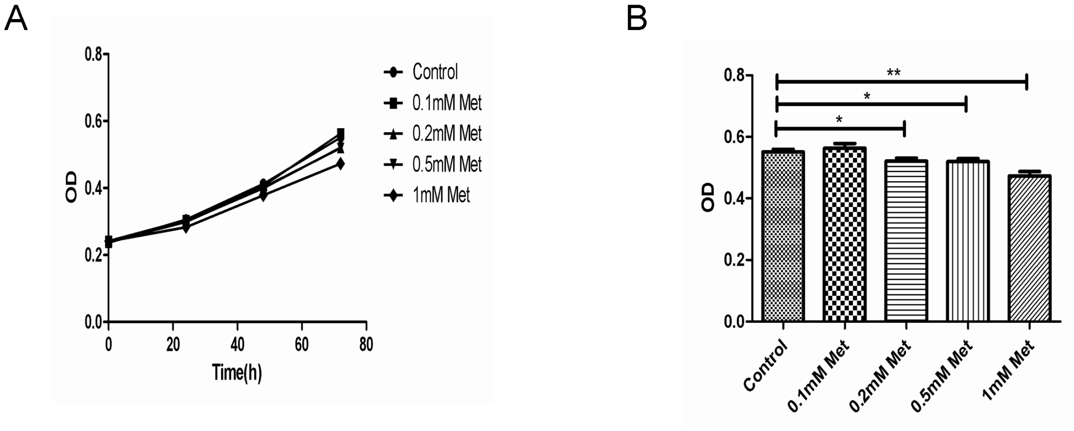

Supplementary Figure 1: GH3 cells were incubated with metformin of lower concentrations. **(A)** GH3 cells were incubated with different doses of metformin (0, 0.1, 0.2, 0.5 and 1 mM) and cell proliferation assay was performed daily from 0 to 72 hours. **(B)** GH3 cells were treated with metformin (0, 0.1, 0.2, 0.5 and 1 mM) for 72 hours, and cell proliferation assay was performed. The data were represented as means ± SEM; n=6. *$P < 0.05$;**$P < 0.01$.

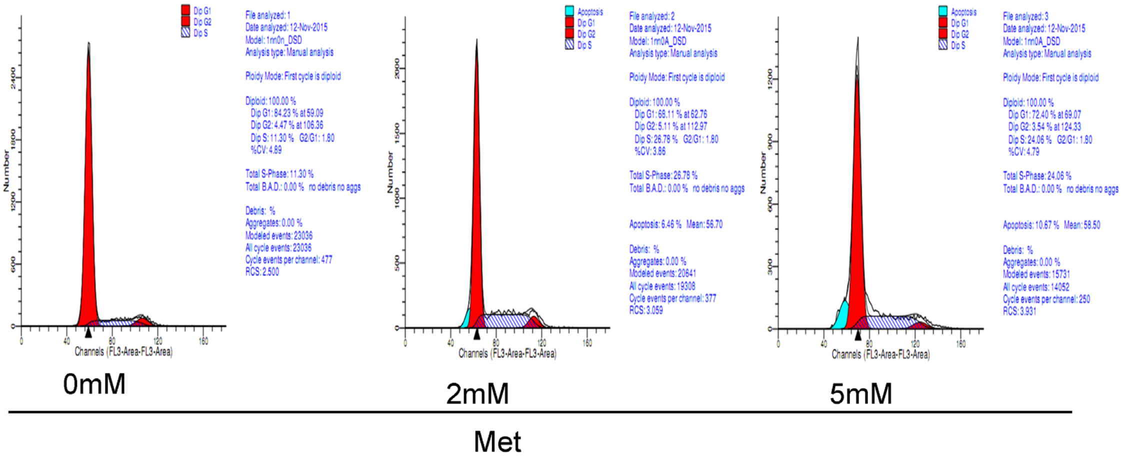

Supplementary Figure 2: GH3 cells were treated with metformin (0, 2 and 5 mM) for 48 h, and cell cycle distributions were analyzed using flow cytometry.

Supplementary Table 1: Clinical characters of 8 GH-PA patients

Case	Age	Gender	Tumor size(cm)	Serum GH (ng/ml)	Serum IGF-1 (ng/ml)	Invasiveness
1	50	F	1.5x1.8x1.9	54.8	1008	No
2	66	F	2.6x2.7x2.0	24.1	713	Yes
3	51	F	2.2x2.4x3.3	15.5	370	Yes
4	37	M	2.9x4.5x3.5	21.9	1013	Yes
5	30	M	1.0x1.2x0.8	39.8	762	No
6	28	F	3.3x2.3x2.0	71.9	859	Yes
7	62	F	0.5x0.7x1.0	26.6	520	No
8	53	M	2.2x2.2x2.4	15.9	764	Yes

M: male; F: female.
